# Safety and Potential Neuromodulatory Effects of Multi-Wall Carbon Nanotubes in Vertebrate and Invertebrate Animal Models In Vivo

**DOI:** 10.3390/ijms262210844

**Published:** 2025-11-08

**Authors:** Valentina Latina, Marzia Soligo, Tatiana Da Ros, Emily Schifano, Marco Guarnieri, Arianna Montanari, Giuseppina Amadoro, Silvana Fiorito

**Affiliations:** 1 Institute of Translational Pharmacology, National Research Council, Via Fosso del Cavaliere 100, 00133 Rome, Italy; valentina.latina80@gmail.com (V.L.); g.amadoro@cnr.it (G.A.); 2 European Brain Research Institute (EBRI), Viale Regina Elena 295, 00161 Rome, Italy; 3 Department of Chemical and Pharmaceutical Sciences, University of Trieste, Via Licio Giorgieri 1, 34127 Trieste, Italy; daros@units.it; 4 Department of Biology and Biotechnologies Charles Darwin, Sapienza University of Rome, Piazzale Aldo Moro 5, 00185 Rome, Italyari.montanari@uniroma1.it (A.M.); 5 Department Clinical Medicine, Sapienza University of Rome, Piazzale Aldo Moro 5, 00185 Rome, Italy

**Keywords:** nanomaterials, carbon nanotubes (CNTs), Multi-Wall Carbon Nanotubes (MWCNTs), in vivo studies, carbon nanotube biocompatibility, carbon nanotube toxicity, Central Nervous System (CNS)

## Abstract

Multi-Wall Carbon Nanotubes (MWCNTs) are under investigation for their use in biomedical applications, especially in neurological diseases, due to their electrochemical properties. Nevertheless, conflicting results have cast doubt on their safety. To advance their translational potential, we evaluated the cytotoxicity of two MWCNT samples in vivo in both vertebrate and invertebrate animal models. Pristine MWCNTs were, in part, used as prepared (MWCNTs), and, in part, annealed at 2400 °C (a-MWCNTs). The two samples differ in their electrochemical properties: MWCNTs are not electro-conductive, while a-MWCNTs are electro-conductive and negatively charged on their surface. We evaluated the effects of both intranasally delivered MWCNTs on several key markers of cell viability in the olfactory bulbs and hippocampus from healthy adult Wistar rats, as well as their impact on lifespan, genotoxicity, oxidative stress, and aging-related functional markers in the nematode *Caenorhabditis elegans*. Neither of the two MWCNT samples was cytotoxic towards neuronal cells in the hippocampus. In olfactory bulbs, only electro-conductive a-MWCNTs interacted with two positively charged mitochondrial proteins: Translocase of Outer Mitochondrial Membrane 20 (TOM20) and Cytochrome C (CytC). In *C. elegans*, neither type of MWCNT affected lifespan or brood size, and cytosolic ROS levels remained unchanged. Notably, treated worms exhibited a significantly delayed aging phenotype. Metallic MWCNTs are biocompatible in living organisms and possess the potential to modulate neural cells functioning in vivo.

## 1. Introduction

Due to the ever-growing interest in their prospective biomedical and/or pharmaceutical applications, the toxicity of Carbon Nanotubes (CNTs) has been deeply investigated since almost their discovery, about three decades ago [1,2]. These nanomaterials, due to their unique combination of mechanical, electrical, chemical, and physical properties, have been positively applied to a wide variety of nanotechnological advances [3]. Nevertheless, in recent years, it has become clear to the scientific community that the toxicity of nanotubes strictly depends on their structural and functional characteristics, which make each nanotube different from the others. The shape, size, number of walls, agglomeration state, surface electrical charges, presence of reactive chemical sites, and/or of different appendages substantially change their biological properties and behavior in vivo, making them more or less cytotoxic [4]. Consequently, the biomedical potential of CNTs needs to be always evaluated in relation to the peculiar and individual properties of these nanostructures that make them more or less cytotoxic. Indeed, an accurate characterization of each nanotube sample is imperative before assessing its potential for biological and/or theranostic purposes. Low water solubility and toxicity can significantly limit the application of CNTs in biomedical sciences, but they can be overcome primarily with functionalization [5]. More recently, CNTs, due to their extraordinary electrical properties, have been mostly explored as innovative brain delivery devices and promising therapeutic tools to treat neurodegenerative diseases and brain injury as well [6,7]. Several studies have highlighted the importance of their electrochemical properties, which are responsible for their effect on the Central Nervous System (CNS) by stimulating and/or modulating neuronal cell functioning [8,9,10,11,12]. Consistently, CNTs have been exploited into the brain as (i) scaffolds to support neuronal growth and enhance the performance of synaptic interfaces [13]; (ii) vectors for intracerebral delivery of nucleic acids to reduce damage and improve recovery following stroke [14,15]; and (iii) platforms to display antigens to ameliorate immune responses against gliomas [16]. Furthermore, due to the high degree of heat that they generate upon exposure to near-infrared radiation, CNTs are useful for photothermal cancer therapy [17,18], in particular of glioblastoma, a deadly brain tumor [19,20,21]. However, even though the advantages of CNTs as innovative medical devices have been largely documented, their clinical use in neuroscience has not yet been completed owing to the high concern raised in relation to their toxicity, biosafety, and biodegradation [12,22]. Concerning their safety profile, CNTs have been shown not to inhibit the capacity of Human Brain Microvascular Endothelial Cells (HBMECs) to form or maintain vessel-like structures [23]. These findings are consistent with other in vitro results showing that carboxylated or amine functionalized CNTs (as opposed to pristine CNTs) do not greatly affect blood vessel integrity [24,25,26,27]. Likewise, no change in cell viability, neuronal morphology, and electrical activity has been found in primary cultured neurons after incubation with CNTs [28], further supporting their high biocompatibility with neuronal cells [29,30,31]. The cytotoxicity of CNT exposure has also been evaluated in vivo in various brain cell types, including microglia [32,33], astrocytes, and neurons [15,33], with conflicting results. For instance, both cytotoxic and cytoprotective properties have been reported following exposure of neuronal cells to carbon nanomaterials [34,35], even though the actual molecular mechanisms governing, at the ultrastructural level, the mutual interactions between these nanomaterials and the neural cells have been only poorly explored [36,37,38]. We investigated the toxic effects of the same two nanotube samples, pristine (MWCNTs) and annealed Multi-Walled Carbon Nanotubes (a-MWCNTs), used for this study, on different cell types. Our findings [39] obtained in vitro with human peripheral blood monocyte derived macrophages have shown not only that the annealed and electroconductive MWCNTs are not toxic but also that such interaction is regulated by their electrochemical properties. Electro-conductive MWCNTs were able to (i) activate macrophage cells without being cytotoxic; (ii) induce reorganization of the actin–cytoskeleton network; and (iii) change the intracellular pH and the mitochondrial membrane potential in a very short time, showing that electro-chemically mediated mechanisms, taking place between cell membranes and electro-conductive Multi-Walled Carbon Nanotubes, could play a key role in the interactions between these nanotubes and cells. Moreover, we also demonstrated [40], in microglia cells from rat brain cortex, that the same electroconductive a-MWCNTs not only were not cytotoxic, thus not inducing cell necrosis or apoptosis, but also impacted microglia cell phenotype and function inducing the transition of microglia from a classical M1 (pro-inflammatory) to alternative M2 (anti-inflammatory) phenotype with production of anti-inflammatory cytokines and the neurotrophic mature Nerve Growth Factor (mNGF). These findings emphasize the role played by electro-conductive CNTs in modulating brain cell behavior.

In addition, confirming our previous in vitro results, we have reported that following intra-nasal delivery in healthy controls and Streptozotocin (STZ)-treated neurodegenerative rats, both properly functionalized MWCNTs gain access in vivo to the CNS via the olfactory route and widely distribute throughout the brain parenchyma. More importantly, confirming our previous results obtained with rat brain microglia, a-MWCNTs were observed to induce a significant increase in mNGF into the hippocampus of diabetic rats, hinting at their possible stimulation of neuroreparative, NGF-based mechanisms [41]. The present study is part of this line of research aimed at demonstrating that a certain type of CNTs, when possessing special electrochemical characteristics, can stimulate/modulate the functioning of brain cells both in vitro and in vivo. Objectives: Based on these encouraging results, we set about better exploring the toxicity and biocompatibility of these two well-characterized (in terms of degree of purity, morphology, structure, nanotexture, and electrochemical properties) MWCNTs in vivo to gain a more complete picture of their biocompatibility and ability to interface with neural cells. To achieve this goal, both vertebrate and invertebrate animal models were used for the study with the aim of providing a broader evaluation of the toxicity of MWCNTs. Both MWCNTs were delivered to healthy adult rats by nasal instillation or administered into the culture media of the worm *Caenorhabditis elegans*, followed by biochemical evaluation of key markers of neuronal homeostasis, along with lifespan and fertility assays. The animal models were chosen because the rat is the animal best suited for intranasal administration and because *C. elegans* has only 20,000 genes, a number surprisingly close to that of humans, but in a much simpler environment to study, and has a short life cycle that allows the effects of a substance to be studied throughout its lifespan. We chose to examine the olfactory bulbs and the hippocampus for different reasons: (i) since these brain areas are in vivo targeted by both MWCNTs after administration via the nasal route in rats [41], and (ii) because we wanted to study the effects of nanotubes on two particularly sensitive brain regions that are severely affected in several neurodegenerative disorders.

## 2. Results

### 2.1. Impact of a-MWCNTs and MWCNTs on Rat Brain Physiology

**Figure 1:**
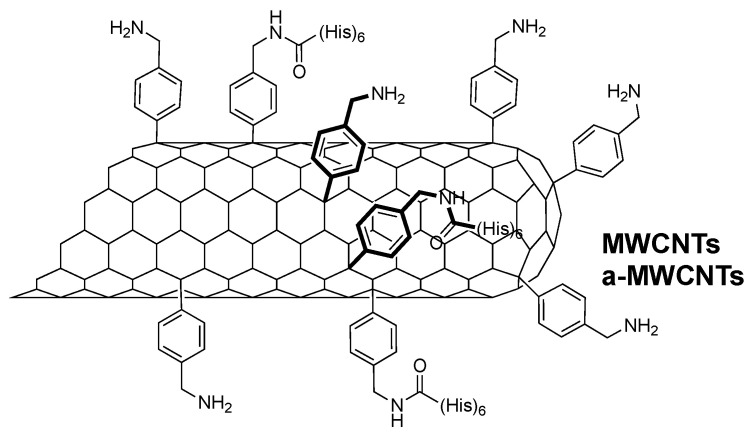
Structure of the two functionalized MWNCTs.

As previously reported [41], MWCNTs were functionalized to improve their water dispersibility and to introduce Histidine tags. The synthesis approach takes advantage of the Tour reaction to introduce some appendages on the tubes, without altering their electrochemical characteristics, with the purpose of not affecting the peculiar conductivity of a-MWCNTs [41]. So, from here on, we will refer to MWCNTs and a-MWCNTs as functionalized derivatives (Figure 1). To evaluate the effect of intranasally delivered MWCNTs on brain physiology, Western blotting analyses followed by semi-quantitative densitometry were carried out on total protein homogenates of both hippocampus and olfactory bulbs from animals of three experimental groups (saline-, MWCNTs, and a-MWCNTs-treated). For probing, we used several commercial antibodies directed against well-established structural and functional markers related to different aspects of brain homeostasis whose expression patterns change under pathological conditions in human brain diseases [42]. We investigated cell-specific markers of differentiated neuronal and glial cells involved in both neuronal development and neurodegeneration (Amyloid Precursor Protein, AβPP), as well as cytoskeleton stability (Microtubule-Associated Protein (MAP) tau), gliosis (Glial Fibrillary Acidic Protein, GFAP), neuronal injury (Ubiquitin carboxy-terminal hydrolase L1, UCHL-1), synapse integrity (SyNaptosome Associated Protein 25, SNAP-25; Alpha-synuclein, α-syn), and mitochondrial function (Translocase of Outer Mitochondrial Membrane 20, TOM20; Cytochrome C, CytC).

**Figure 2:**
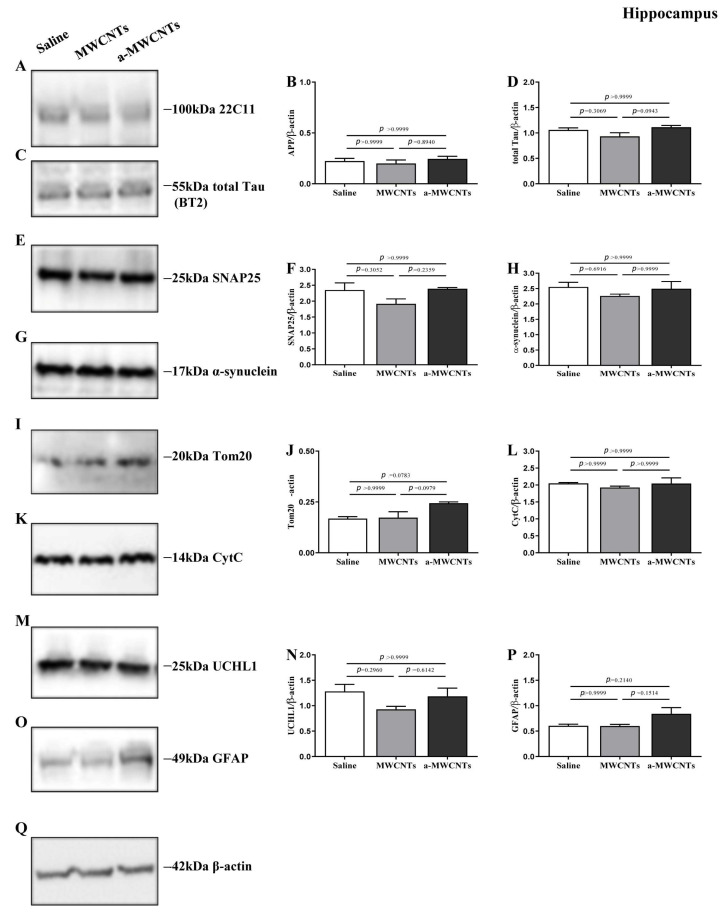
Effect of nasal delivery of MWCNTs and a-MWCTNs on the expression of both neuronal and glial specific markers in the hippocampi of Wistar rats. (**A**,**C**,**E**,**G**,**I**,**K**,**M**,**O**) Representative images of Western blotting analysis (*n*  =  10 animals per group, 5 males and 5 females for each experimental condition) carried out on whole protein lysates of the hippocampi of animals from the three experimental groups (saline-, MWCNTs- and a-MWCNTs-treated) with 22C11 (66–81aa of N-terminus of APP), BT2 (194–198aa of full-length tau protein), SNAP25, α-syn, Tom20, CytC, UCHL-1, and GFAP antibodies, as indicated alongside the blots. Legends on the right side indicate the molecular weight (kDa) of bands calculated from the migration of standard proteins. (**B**,**D**,**F**,**H**,**J**,**L**,**N**,**P**) Histograms show the semi-quantitative densitometry of the intensity signals in immunoreactivity bands by normalization with β-actin (**Q**) level used as loading control. *p* < 0.05 is accepted as statistically significant (one-way ANOVA followed by Bonferroni’s post hoc test for multiple comparisons among more than two groups.

The hippocampus and olfactory bulbs were chosen since these brain areas are (i) in vivo targeted by both MWCNTs after administration via the nasal route in rats [41] and (ii) are severely affected in several neurodegenerative disorders, to counter which nanoparticle-based drug delivery strategy is currently employed [36,43,44,45]. As shown in Figure 2, in the hippocampus, the endogenous steady-state expression levels of analyzed markers were not significantly changed among the three different experimental groups (one-way ANOVA followed by Bonferroni’s post hoc test; *p* > 0.9999), suggesting that nasal inhalations of MWCNTs and a-MWCNT are not harmful to the cellular population of this cerebral area.

**Figure 3:**
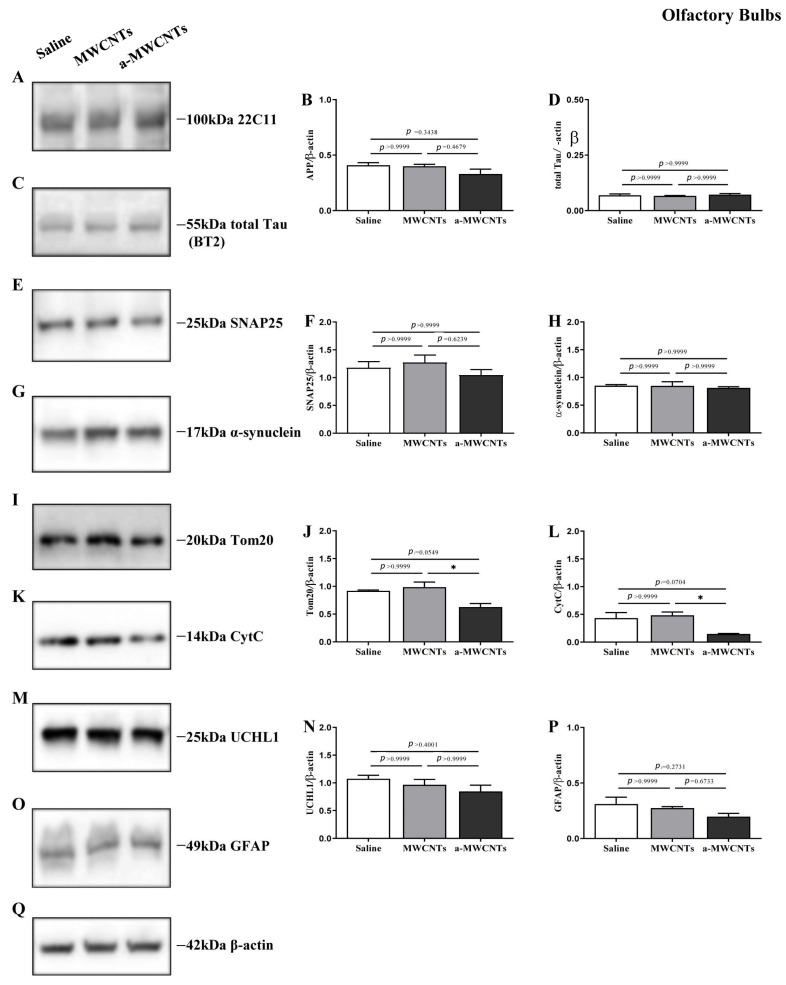
Effect of nasal delivery of MWCNTs and a-MWCTNs on the expression of both neuronal and glial specific markers in the olfactory bulbs of Wistar rats. (**A**,**C**,**E**,**G**,**I**,**K**,**M**,**O**) Representative images of Western blotting analysis (*n*  =  10 animals per group, 5 males and 5 females for each experimental condition) carried out on whole protein lysates of the olfactory bulbs of animals from the three experimental groups (saline-, MWCNTs- and a-MWCNTs-treated) with 22C11 (66–81aa of N-terminus of APP), BT2 (194–198aa of full-length tau protein), SNAP25, α-syn, Tom20, CytC, UCHL-1, and GFAP antibodies, as indicated alongside the blots. Legends on the right side indicate the molecular weight (kDa) of bands calculated from the migration of standard proteins. (**B**,**D**,**F**,**H**,**J**,**L**,**N**,**P**) Histograms show the semi-quantitative densitometry of the intensity signals in immunoreactivity bands by normalization with β-actin (**Q**) level used as loading control. *p* < 0.05 is accepted as statistically significant (one-way ANOVA followed by Bonferroni’s post hoc test for multiple comparisons among more than two groups). * *p* < 0.05.

On the contrary, only the inhalation of a-MWCNTs provoked into the olfactory bulbs a significant reduction in the expression of TOM20 and CytC immunoreactivity (one-way ANOVA followed by Bonferroni’s post hoc test; * *p* < 0.05 a-MWCNTs vs. MWCNTs), hinting at their potential modulatory actions on mitochondrial physiology of this specific cerebral region (Figure 3). Nevertheless, no change was detected for the other analyzed markers among the three different experimental groups (one-way ANOVA followed by Bonferroni’s post hoc test; *p* > 0.9999). Our findings show that intranasal delivery of both MWCNTs and a-MWCNTs does not alter any of the specific cell-type markers of brain homeostasis in the hippocampi of healthy adult rats but induces a significant reduction in the two key mitochondrial proteins, Cytochrome C and Translocase of the Outer Membrane complex, into animals olfactory bulbs.

### 2.2. Impact of a-MWCNTs and MWCNTs on C. elegans Healthspan

The nematode *Caenorhabditis elegans* is a well-established model in the biosafety assessment of nanomaterials [46]. Indeed, *C. elegans* has several remarkable features, including a short life cycle, ease of handling, a small and transparent body, and a well-defined genetic background.

**Figure 4:**
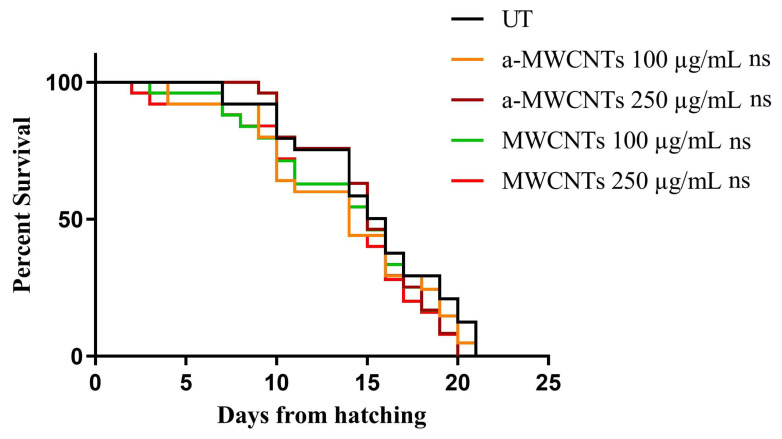
Effect of different concentrations of MWCNTs and a-MWCTNs on *C. elegans* lifespan. Kaplan–Meier survival plot of N2 worms treated with different concentrations of Carbon Nanotubes. UT, untreated animals; n = 90 for each replicate. ns: not significant (one-way ANOVA, followed by the Bonferroni post-test).

**Figure 5:**
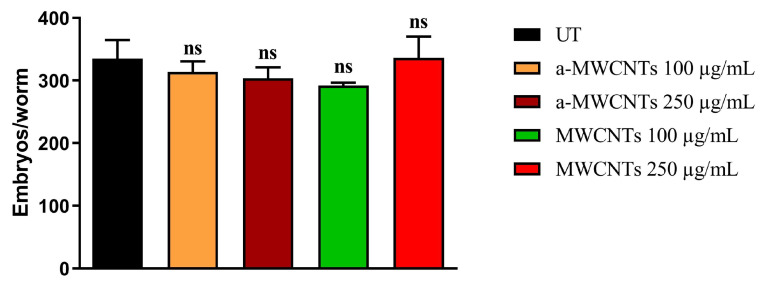
Effect of different concentrations of MWCNTs and a-MWCTNs on *C. elegans* fertility. Evaluation of the brood size of animals treated with a-MWCNTs or MWCNTs. Average embryo production per nematode worm, treated as indicated. UT, untreated animals; ns, not significant (one-way ANOVA, followed by the Bonferroni post-test).

To evaluate the in vivo toxicity of MWCNTs and a-MWCNTs, a lifespan assay was performed. The animals treated with 10 µg mL^−1^ and 50 µg mL^−1^ of both types of Carbon Nanotubes presented an almost identical survival of the untreated population. As shown in Figure 4, similar results were also obtained at higher doses of tubes (100 µg mL^−1^ and 250 µg mL^−1^), with no significant difference between controls and the treated population (one-way ANOVA, followed by the Bonferroni post-test; *p* > 0.9999). In the case of genotoxicity assessment, the evaluation of the progeny derived from animals treated with MWCNTs or a-MWCNTs was performed (Figure 5). The fertility analysis demonstrated that the brood size of the worms treated with the two higher concentrations of nanotubes (100 or 250 µg mL^−1^) was identical to the control group (one-way ANOVA, followed by the Bonferroni post-test; *p* > 0.9999), highlighting the lack of genotoxicity of these nanomaterials. The results obtained in *C. elegans* confirm that neither of the MWCNT samples affects the life span and does not induce genotoxicity in vivo in the invertebrate model.

**Figure 6:**
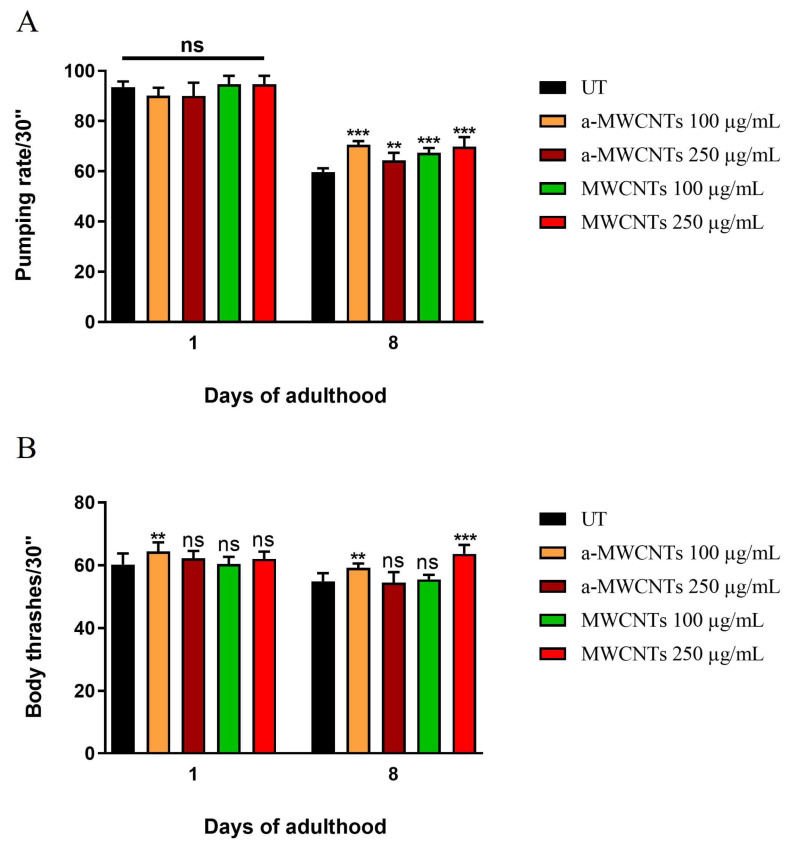
Functional aging markers in *C. elegans* following exposure to MWCNTs. (**A**) Pharyngeal pumping rate and (**B**) locomotion activity recorded in 1- and 8-day-old adults grown in the presence of MWCNTs or a-MWCNTs at the indicated concentrations. Data are expressed as mean ± SEM of three independent experiments. Statistical analysis: one-way ANOVA with Bonferroni’s post hoc test (** *p* < 0.01, *** *p* < 0.001 vs. untreated controls; ns: not significant).

Pharyngeal pumping and locomotion activity were evaluated at early adulthood and during aging (Figure 6). At day 1, all MWCNT-treated groups showed equal or slightly higher activity than controls, confirming the absence of early neuromuscular toxicity. At day 8, treated animals maintained significantly higher pumping and body bend rates than untreated worms, demonstrating a slower age-related functional decline. Notably, locomotion analysis revealed a dose-dependent improvement, with animals exposed to the 250 µg/mL MWCNT concentration displaying the greatest increase in body bending frequency, further supporting a pro-healthy aging effect.

**Figure 7:**
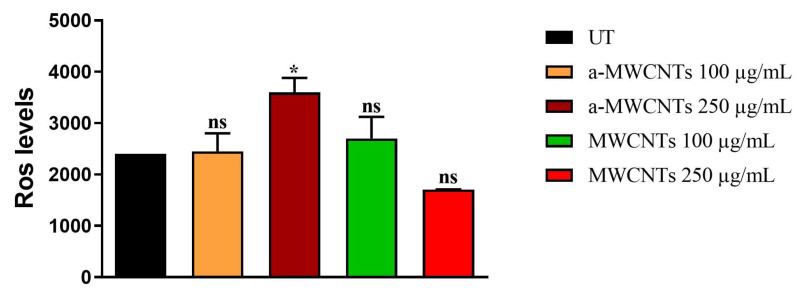
Cytosolic ROS levels in *C. elegans* exposed to MWCNTs. Quantification of H_2_DCF-DA fluorescence in 8-day-old adult worms continuously exposed to pristine (MWCNT) or annealed (a-MWCNT) nanotubes at 100 and 250 µg/mL. Data are expressed as mean ± SEM of three independent experiments. One-way ANOVA followed by Bonferroni’s post hoc test (* *p* < 0.05; ns: not significant).

Moreover, cytosolic Reactive Oxygen Species (ROS) levels were measured in 8-day-old worms after continuous exposure to MWCNTs. As shown in Figure 7, no significant differences were observed between untreated controls and MWCNT-treated groups at both 100 and 250 µg/mL. A slight increase was detected only in worms exposed to 250 µg/mL a-MWCNTs (*p* < 0.05); however, without a dose-dependent trend, indicating no relevant oxidative stress induction.

## 3. Discussion

Our results show that (i) intranasal delivery of both MWCNTs and a-MWCNTs does not alter any of the analyzed specific cell-type markers of brain homeostasis in the hippocampi of healthy adult rats; (ii) a significant reduction in the two key mitochondrial proteins, CytC and TOM20, is detected in the animals’ olfactory bulbs following exposure to a-MWCNTs, whereas MWCNTs are contextually ineffective; (iii) health span and oxidative stress are not affected by either a-MWCNTs or MWCNTs in the *C. elegans* invertebrate model. It is noteworthy that olfactory bulbs are easily reached by Carbon Nanotubes quite immediately after their acute delivery via inhalation, being detectable in this rat brain area 24 h post-treatment. On the contrary, a chronic (three-day) treatment regimen is necessary to let them reach the deeper structures in the basal forebrain, striatum, hypothalamus, hippocampus, and cortex [41]. Thus, it is conceivable that the highest quantity of nanotubes that gain access to the olfactory system immediately after their nasal instillation can be responsible for the greater effect we detected in the olfactory bulbs rather than in the hippocampus. Nevertheless, it must be kept in mind that a limitation of the study may be related to the minimal differences in the amount of substance inhaled due to reasons independent of the administration method, such as anatomical or functional differences in the nasal cavities of rats.

The finding that only annealed and electro-conductive a-MWCNTs turned out to induce a significant decrease in CytC and TOM20, whereas MWCNTs were contextually ineffective, was unexpected because the latter was supposed to be more capable of binding to chemical groups and interacting with cell structures due to the numerous reactive sites on their surface (not being annealed), giving rise to more interactions with the cell structures. CytC is a redox-active, highly positively charged mitochondrial protein [47]. In a similar way, the Translocase of the Outer Membrane complex—which is bound to the mitochondrial Outer Membrane through the NH2-terminal Trans Membrane Domain (TMD)—also carries a net positive charge within the five residues of its COOH-terminal flanking region [48]. We hypothesized that the decreased detection of the two mitochondrial molecules, as detected by Western blot analyses in olfactory bulbs of rats administered annealed-MWCNTs, can be mainly ascribed, as already observed in our previous experiments, to the sorption of divalent cations on their surface because of the distribution of electrons on a more homogeneous surface following the annealing process. Consistent with this hypothesis, we have previously demonstrated that the same electrically conductive samples of MWCNTs, due to the sorption of Ca^2+^ ions on their surface, affected Ca^2+^ ion balancing between extra- and intracellular environments in the rats’ electrically sensitive neuronal cells, induced changes on Ca^2+^ ion-dependent cellular junctions in rat adenocarcinoma cells and affected platelet aggregation, behaving as the calcium chelator ethylene glycol tetra-acetic acid [49]. Relevantly, and in line with our previous results showing electrochemical and anti-inflammatory properties of a-MWCNTs [41], many pieces of evidence suggest that metal cations, such as Ca^2+^ and Cu^2+^ ions, reduce the pro-inflammatory activation of microglia and astrocytes in vitro [50,51]. Therefore, the interaction/binding of the highly positively charged CytC and TOM20 to the surface of electron-rich a-MWCNTs is more likely indicative of their decreased detection following in vivo treatment with a-MWCNTs, than of their cytotoxic effect towards cellular components. Cytochrome C is physiologically localized in the mitochondrial intermembrane space and regulates intracellular processes that are crucial for cell viability [52]. On one hand, cytochrome C is indispensable for metabolic energy production, being an important component of the respiratory chain acting as an electron carrier into the mitochondrial Electron Transport Chain (ETC), which is coupled with the production of Adenosine Tri Phosphate (ATP) and also of deleterious Reactive Oxygen Species (ROS) [53]. On the other hand, cytochrome C plays a key role as an activator of programmed cell death in triggering mitochondria-induced apoptotic signal transduction pathway [54]. Neuronal populations requiring high levels of energy for their normal functioning strictly depend on mitochondrial activity, which makes them very vulnerable to ROS generation and oxidative stress, and, then, the maintenance of the brain mitochondrial homeostasis is of paramount importance to counteract the onset and progression of several neurodegenerative disorders [55]. Nevertheless, even though any change in mitochondrial abundance of CytC could be potentially harmful to neurons, the controlled and sub-toxic depletion of this molecule has been proven to be sufficient to render cells incapable of triggering apoptosis. In addition, the residual CytC concentration seems to be sufficient for sustaining cellular respiration (sometimes compensated for enhanced glycolysis) when there is only low ATP synthase activity [47,48]. From this point of view, the sub-toxic downregulation in the steady-state expression level of CytC induced by a-MWCNTs might be suggestive, from a translational point of view, for potential future therapeutic uses in neurodegenerative diseases as neuroprotective agents and metabolic agents by decreasing the metabolic rate (i.e., ROS production) and/or preventing the apoptosis activation under several brain-related pathological conditions.

The Translocase of the Outer Membrane is a pore-forming complex that acts as a dynamic gatekeeper in guiding and importing pre-proteins with specific mitochondria-targeting sequences. Interestingly, TOM20, a component of this multi-subunit system, is the main receptor responsible for the uptake and translocation of the toxic Amyloid β peptide(s) (Aβ) produced from APP proteolysis by its processing enzymes (β-secretase and γ-secretase) and accumulating into mitochondria of vulnerable brain regions from individuals affected by Alzheimer’s Disease (AD) and preclinical mouse models [56]. In this context, the downregulation of TOM20 might be exerting a protective role by limiting the buildup of Aβ into the brain mitochondria under pathological AD-like conditions, thus paving the way for the use of MWCNTs as possible therapeutic agents to counteract this age-related neurodegenerative disorder.

Consistent with these findings on rat brain tissue, *C. elegans* experiments confirmed the absence of toxicity, as treated animals with 100 and 250 µg mL^−1^ showed survival rates like controls, and no genotoxic effects were observed. Moreover, MWCNTs did not affect oxidative stress responses, and treated worms exhibited a slight delay in age-related functional decline.

The literature data on the toxicity of MWCNTs are contradictory and give inconsistent results due to the MWCNTs’ characteristics that can influence their bioactivity in living cells both mechanically and chemically. The size (length and/or diameter), functionalization, purification, and even the experimental method can affect their biocompatibility and cytotoxicity. A recent study, showing the high cytotoxicity of MWCNTs with a large diameter (>50 nm), as well as moderate and low cytotoxicity of MWCNTs with a small diameter (<20 nm), suggested that the diameter of MWCNTs considerably contributes to their cytotoxicity [57]. Since the diameter of MWCNTs used for this study is in the range between 10 and 20 nm, this is consistent with their low cytotoxicity. As it concerns mitochondrial toxicity, it has been reported that MWCNTs conjugated to a mitochondria-targeting peptide sequence exhibited a high level of nanotube accumulation in mitochondria of murine macrophages and HeLa cells without showing any significant cytotoxicity [58]. More recently, we demonstrated the in vitro viability of human peripheral blood mononuclear cells exposed to MWCNT conjugates, showing that the conjugates do not affect cell mitochondrial function [59]. On the contrary, MWCNTs were also shown to cause mitochondrial dysfunction leading to mitophagy in exposed human bronchial epithelial cells [60], and it was suggested that SWCNTs and MWCNTs likely damage brain tissue mitochondria by increasing oxidative stress and possibly activating the apoptosis pathway as well as other pathways of cytotoxicity [60].

We are aware that further studies to investigate the toxicity and biocompatibility of MWCNTs, and especially a long-term study in vivo, have to be performed to verify and confirm our hypothesis and to validate the absence of neurotoxicity before any biomedical application can be proposed.

## 4. Materials and Methods

### 4.1. Carbon Nanotubes

Pristine Multi-Walled Carbon Nanotubes (MWCNTs) (CRMD, Orléans, France) were synthesized, as previously described by a regular Catalyst-assisted Chemical Vapor Deposition technique (CCVD) [44]. The MWCNTs obtained were in part used as-prepared (MWCNTs), and in part annealed at 2400 °C under an argon atmosphere (a-MWCNTs) to be purified. The outer diameters of the produced tubes were in the 10–20 nm range [61], and the average number of walls was ~15. The degree of purity and the morphology, structure, and nanotexture of both MWCNT samples were evaluated by X-ray diffraction (XRD), X-ray Photoelectron Spectroscopy (XPS), Transmission Electron Microscopy (TEM), and correspond to what was previously reported [39], showing that the annealing treatment eliminated any trace of catalysts, as well as of reactive chemical sites, and reduced the content of oxygen, while maintaining their integrity, their structure, and their shape.

### 4.2. Carbon Nanotube Functionalization

The functionalization of the tubes was performed by the Tour reaction [62] following the procedure already reported [41], where both MWCNTs and a-MWCNTs were allowed to react with 4-(N-Boc-aminomethyl) aniline (Sigma Aldrich, St. Louis, MO, USA)and isopentyl nitrite, and later the Boc protection was removed by HCl treatment. The ammonium functionalities were deprotonated by DIEA and partially coupled using a hexa-His tag. The latter allowed the detection of the conjugated nanotubes in the tissues, using specific antibodies. All the characterization of the nanotubes has already been reported [41].

### 4.3. Electrochemical Characterization

The electrochemical characterization was carried out through the measurement of Cyclic Voltammetry (CV) and of the Open Circuit Potential (OCP) as previously described [41]. The electrochemical characterization was performed in a glass cell equipped with a platinum spiral as a counter electrode and a saturated KCl calomel reference electrode (SCE). The CNT film was deposited as a 6 mm diameter disk, by drop-casting 40 mL of a 1 mg mL^−1^ sonicated suspension of CNTs in PBS on an Indium Tin Oxide (ITO)-coated glass slide. The 40 mL were deposited in 4 steps of 10 mL each (confining the drop within an O-ring), followed by drying under vacuum for 45 min. Such a stepwise deposition-drying procedure allows obtaining a more homogeneous and tightly adsorbed film onto the ITO surface [63]. The Open Circuit Potential (OCP) and Cyclic Voltammetry measurements were performed at RT in a PBS electrolyte solution by using a Biologic potentiostat SP300 instrument. The current–potential curves of the two types of MWCNTs in particular showed that a-MWCNTs had a higher electrical performance and capacitance with respect to as-prepared MWCNTs. Moreover, OCP studies, performed on MWCNT films with increasing concentrations of three ions, namely Ca^2+^, Cu^2+^ and Zn^2+^, showed that copper ions had a higher affinity towards a-MWCNTs, very similar to the case of calcium ions, thus confirming the previous observed cationic exchange ability of our highly electro-conductive a-MWCNT sample [49]; the ability of annealed MWCNTs to adsorb divalent positively charged ions on their surface due to oxidation processes that cause the ripristinization of the surface, removing the majority of the defects, allow a better capability to conduct electrons. This, as a consequence, permits an increased capacity of cationic exchange [64].

### 4.4. Animals Handling and Intranasal Drug Delivery

Male Wistar rats (350–400 g) were purchased from Charles River Laboratories s.r.l. (Calco, Lecco, Italy). Rats were weighed, ears and tails were tagged, and they were housed in groups of two to three per cage with standard food and water available ad libitum. The animal room had a controlled 12 h light cycle (lights on at 07:00 h), lux level (on average 100 lux), temperature (21 ± 1 °C), and relative humidity (50 ± 5%). Rats were arbitrarily divided into three experimental groups as follows: 10 (5 male and 5 female) rats treated with saline (saline), 10 (5 male and 5 female) rats treated with MWCNTs (MWCNTs-treated), 10 (5 male and 5 female) rats treated with a-MWCNTs (a-MWCNTs-treated). All treated and untreated rats were included in the study. Rats were slightly anesthetized with isoflurane and exposed to intranasal administrations of MWCNTs, a-MWCNTs, or saline once a day for three days, as we previously reported [41], without a control for potential confounders. Rats received 72 μg of MWCNTs per day, dispersed in the solution (two applications of 9 μL for each nostril, for a total of 36 μL) for each day of treatment (maximum amount of CNTs: 216 μg per rat). Animal care procedures were conducted in conformity with the legislation for the protection of animals used for scientific purposes provided by the relevant Italian law and European Union Directive (Italian Legislative Decree 26/2014 and 2010/63/EU). The current study was conducted by investigating brains from healthy rats, which were part of a previous study [41] approved by the Veterinary Department of the Ministry of Health (Authorization n. 192/2015-PR, 30 March 2015). All adequate measures were taken to minimize animal pain or discomfort, and it was not necessary to establish human endpoints, as no adverse effects of the treatment were expected. Experimental researchers were blinded in the experimental conditions throughout the data analysis.

### 4.5. Protein Cell Lysates Preparation

The day after the end of intranasal treatments, rats were deeply anesthetized with isoflurane, sacrificed by decapitation, and the brain tissues of interest (hippocampus and olfactory bulbs) were rapidly dissected and frozen until use. Samples were homogenized, using a glass tissue grinder, in 50 volumes of ice-cold Hepes 1% NP40 buffer (20 mM Hepes KOH pH 7.9, 150 mM NaCl, 0.1 mM EDTA, 0.1 mM EGTA 1% (*v*/*v*) NP40), and the resulting crude homogenates were sonicated with a Sonics Vibra Cells (Sonics & Materials, Inc., Newtown, CT, USA). Total protein content was determined using the Bio-Rad DC Protein Assay Kit (Bio Rad, Hercules, CA, USA) according to the manufacturer’s instructions.

### 4.6. Western Blot Analysis and Semi-Quantitative Densitometry

Equal amounts of protein extracts (70 µg) were size-fractionated by SDS-PAGE Bis-Tris gel 4–12% (Bolt, Invitrogen, Thermo Fisher Scientific, Waltham, MA, USA) and transferred to nitrocellulose membranes (0.2 μm, GE healthcare, Little Chalfont, UK), as previously reported [65]. The filters were blocked in TBS-T containing 4% non-fat dried milk for 1 h at room temperature or overnight at 4 °C. Proteins were visualized using appropriate primary antibodies. All primary antibodies were diluted in TBS and incubated with the nitrocellulose blot overnight at 4 °C. Incubation with secondary peroxidase-coupled anti-mouse and anti-rabbit was performed by using the ECL system (Thermo Scientific SuperSignal West Pico (Waltham, MA, USA), 34080; Amersham (Little Chalfont, Buckinghamshire, UK) ECL Prime, RPN2232). β-actin was used as an internal control of protein loading, and semi-quantitative densitometric analysis was carried out by using Image J 1.4 (http://imagej.nih.gov/ij/ (accessed on January 2020)). For quantification, we measured the band intensity by using a signal in the linear range. The following antibodies were used: anti-Amyloid Precursor Protein (AβPP) 22C11 (66–81aa of N-terminus), mouse, APP-MAB348, Chemicon (Millipore, CA, USA); Microtubule Associated Protein tau antibody (BT2), mouse, MN1010, ThermoFisher Scientific; GFAP antibody (2E1), mouse, sc-33673, Santa Cruz (Dallas, TX, USA); anti-SNAP25 antibody (clone SMI 81), mouse, 836301, BioLegend (San Diego, CA, USA); anti α-synuclein antibody (clone 42), mouse, 610786 BD, Transduction Laboratories (Franklin Lakes, NJ, USA); Ubiquitin carboxy-terminal hydrolase L1 antibody (UCHL-1) and antibody (clone C-4), mouse, sc-271639, Santa Cruz; TOM20 antibody (FL-145), rabbit, sc-11415, Santa Cruz; CytC antibody, rabbit, 4272, Cell Signaling (Danvers, MA, USA); anti-β-actin antibody, mouse, S3062, Sigma-Aldrich; anti-mouse IgG (whole molecule)-Peroxidase antibody, A4416, Sigma-Aldrich (St. Louis, MO, USA); anti-rabbit IgG (whole molecule)-Peroxidase antibody, A9169, Sigma-Aldrich (St. Louis, MO, USA).

### 4.7. C. elegans Lifespan and Fertility Assays

The *C. elegans* strain used in this study was wild-type Bristol N2. Nematodes were cultivated at 16 °C on nematode growth medium (NGM) and fed with heat-killed *Escherichia coli* OP50. In total, 60 μL of heat-killed culture was spread on 3.5 cm diameter NGM plates, and 100 μL of different concentrations of MWCNTs and a-MWCNTs were added separately. Heat-killed OP50 cells were prepared as already described [66]. The lifespan assay was conducted on synchronous nematodes prepared as described in [67] on NGM spread with heat-killed *E. coli* OP50 (Caenorhabditis Genetics Center, University of Minnesota, Minneapolis, MN, USA) and different concentrations of MWCNTs or a-MWCNTs. Nematodes grown on heat-killed *E. coli* OP50 were taken as controls. Lifespan analysis was performed at 16 °C, and worms were transferred daily to new plates seeded with fresh lawns. They were scored as dead when they no longer responded to a gentle touch with a platinum wire. At least 90 nematodes per condition were used in each experiment. All lifespan assays were performed in triplicate.

For fertility assays, synchronous worms were incubated at 16 °C on NGM plates seeded with heat-killed *E. coli* OP50 and supplemented with 100 or 250 µg mL^−1^ of MWCNTs or a-MWCNTs, as indicated, allowing for embryo laying. Animals were transferred onto new plates every day, and the number of progenies was counted until the mother worms became infertile. Experiments were performed in triplicate.

### 4.8. Aging Markers Analysis in C. elegans

Pharyngeal pumping rate and locomotion capability were evaluated in 1-day- and 8-day-old adult C. elegans continuously exposed to MWCNTs from hatching. Animals were observed under a SZ30 stereomicroscope (Leica, Wetzlar, Germany), and the number of pharyngeal pumps and body bends was recorded over a 30 s interval for each worm.

### 4.9. Reactive Oxygen Species Evaluation in C. elegans

Intracellular Reactive Oxygen Species (ROS) levels were quantified in 8-day-old worms previously exposed to different concentrations of MWCNTs during larval development. Animals were collected, rinsed thoroughly in M9 buffer to remove residual bacteria, and incubated with the ROS-sensitive fluorescent probe 2′,7′-dichlorodihydrofluorescein diacetate (H_2_DCF-DA; Sigma-Aldrich, Sigma-Aldrich (St. Louis, MO, USA), #287810-100MG).

After 1 h of incubation at 20 °C under gentle shaking and protected from light, fluorescence was recorded using a GloMax^®^ Multidetection System plate reader (Promega, Madison, WI, USA), with excitation set at 485 nm and emission at 520 nm. ROS levels were calculated by normalizing fluorescence intensity to the number of worms per sample.

### 4.10. Data Management and Statistical Analysis

Data were expressed as means ± standard error of the mean (S.E.M.) and were representative of at least three separate experiments (*n* = independent experiments). Statistically significant differences were calculated by one-way analysis of variance (ANOVA) followed by Bonferroni’s post hoc test for multiple comparisons among more than two groups (GraphPad Prism 9.0 software, GraphPad Software Inc., La Jolla, CA, United States). *p* < 0.05 was accepted as statistically significant (* *p* < 0.05; ** *p* < 0.01; *** *p* < 0.0005; **** *p* < 0.0001). The sample size was estimated on the basis of our previously published experiments [41]. An “a priori” estimation to compute the required sample size by a given α power and effect size was carried out by G*Power statistical power analysis (version 3.1.9.4). All statistical analyses were performed using GraphPad Prism 9 software.

## 5. Conclusions

Based on the above considerations, we hypothesize that cation interaction with a-MWCNTs may influence metal homeostasis involved in cell signaling, leading to intracellular decreases in mitochondrial markers CytC and TOM20. This suggests a modulation of their function rather than cytotoxicity. Consistent with this, *C. elegans* experiments confirmed the absence of toxicity, as treated animals with 100 and 250 µg mL^−1^ showed survival rates like controls, and no genotoxic effects were observed. Overall, these findings, confirming our previous results, support the potential of metallic electroconductive MWCNTs to selectively modulate neural cell functions via their electrochemical properties, targeting whole cells or specific structures like mitochondria, without causing cell death. However, further molecular-level studies are necessary to understand their interactions with brain cells before safe application in nanomedicine for CNS degenerative diseases.

## Data Availability

The data presented in this study are available on request from the corresponding author. The data are not publicly available due to privacy restrictions. All the data obtained by Western blotting supporting this article have been analyzed using Image J 1.4 (http://imagej.nih.gov/ij/ (accessed on 1 January 2020)) and Graph Pad (GraphPad Prism 9.0 software, GraphPad Software Inc., La Jolla, CA, USA) software, and have been included in the figures. The characterizations of the MWCNTs used in this study have been previously reported in Soligo et al. [41], Distribution in the brain and possible neuroprotective effects of intranasally delivered Multi-Walled Carbon Nanotubes, Nanoscale Adv. 2021, 3, 418–431 and its supplementary information file. DOI https://doi.org/10.1039/D0NA00869A.
